# Creation of a structured molecular genomics report for Germany as a local adaption of HL7’s Genomic Reporting Implementation Guide

**DOI:** 10.1093/jamia/ocad061

**Published:** 2023-04-20

**Authors:** Caroline Stellmach, Julian Sass, Bernd Auber, Martin Boeker, Thomas Wienker, Andrew J Heidel, Manuela Benary, Simon Schumacher, Stephan Ossowski, Frederick Klauschen, Yvonne Möller, Rita Schmutzler, Arsenij Ustjanzew, Patrick Werner, Aurelie Tomczak, Thimo Hölter, Sylvia Thun

**Affiliations:** Core Facility Digital Medicine and Interoperability, Berlin Institute of Health (BIH) at Charité - Universitätsmedizin Berlin, Berlin, Germany; Core Facility Digital Medicine and Interoperability, Berlin Institute of Health (BIH) at Charité - Universitätsmedizin Berlin, Berlin, Germany; Department of Human Genetics, Hannover Medical School, Hannover, Germany; Fakultät für Medizin, Technische Universität München, Munich, Germany; Emeritus Ropers, Max Planck Institute for Molecular Genetics, Berlin, Germany; IT Department, Universitätsklinikum Jena, Jena, Germany; Core Unit Bioinformatics, Berlin Institute of Health (BIH) at Charité - Universitätsmedizin Berlin, Berlin, Germany; Medical Data Integration Center (MeDIC), Universitätsklinikum Köln, Cologne, Germany; Institute of Medical Genetics and Applied Genomics, University of Tübingen, Tübingen, Germany; Institut für Pathologie, Charité - Universitätsmedizin Berlin, Berlin, Germany; Pathologisches Institut, Ludwig-Maximilians-Universität München, Munich, Germany; Berlin Institute for the Foundations of Learning and Data (BIFOLD), Berlin, Germany; Center for personalized medicine (ZPM), Universitätsklinikum Tübingen, Tübingen, Germany; Center Familial Breast and Ovarian Cancer, National Center of Familial Tumor Diseases and Center of Integrated Oncology, Universitätsklinikum Köln, Cologne, Germany; Institut für Medizinische, Biometrie, Epidemiologie und Informatik Mainz, Universitätsmedizin der Johannes Gutenberg-Universität Mainz, Mainz, Germany; MOLIT Institut gGmbH, Heilbronn, Germany; Liver Cancer Centre Heidelberg, Institute of Pathology, Heidelberg University Hospital, Heidelberg, Germany; Core Facility Digital Medicine and Interoperability, Berlin Institute of Health (BIH) at Charité - Universitätsmedizin Berlin, Berlin, Germany; Core Facility Digital Medicine and Interoperability, Berlin Institute of Health (BIH) at Charité - Universitätsmedizin Berlin, Berlin, Germany

**Keywords:** sequencing data, HL7^®^, Fast Healthcare Interoperability Resources, FHIR^®^, Implementation Guide, molecular genomics report, interoperability

## Abstract

**Objective:**

The objective was to develop a dataset definition, information model, and FHIR^®^ specification for key data elements contained in a German molecular genomics (MolGen) report to facilitate genomic and phenotype integration in electronic health records.

**Materials and Methods:**

A dedicated expert group participating in the German Medical Informatics Initiative reviewed information contained in MolGen reports, determined the key elements, and formulated a dataset definition. HL7’s Genomics Reporting Implementation Guide (IG) was adopted as a basis for the FHIR^®^ specification which was subjected to a public ballot. In addition, elements in the MolGen dataset were mapped to the fields defined in ISO/TS 20428:2017 standard to evaluate compliance.

**Results:**

A core dataset of 76 data elements, clustered into 6 categories was created to represent all key information of German MolGen reports. Based on this, a FHIR specification with 16 profiles, 14 derived from HL7^®^’s Genomics Reporting IG and 2 additional profiles (of the *FamilyMemberHistory* and *RiskAssessment* resources), was developed. Five example resource bundles show how our adaptation of an international standard can be used to model MolGen report data that was requested following oncological or rare disease indications. Furthermore, the map of the MolGen report data elements to the fields defined by the ISO/TC 20428:2017 standard, confirmed the presence of the majority of required fields.

**Conclusions:**

Our report serves as a template for other research initiatives attempting to create a standard format for unstructured genomic report data. Use of standard formats facilitates integration of genomic data into electronic health records for clinical decision support.

## Graphical Abstract

**Figure:**
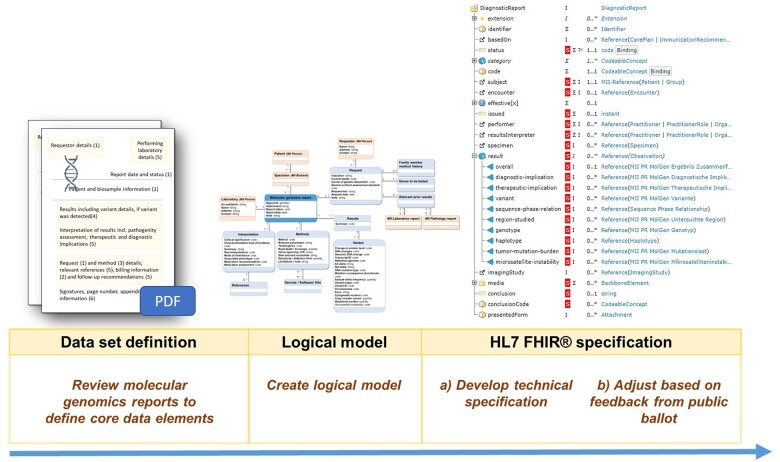

## OBJECTIVE

This study aimed at creating a dataset definition and information model that comprises the key informational elements present in a German MolGen report—defined based on the review of example reports from several German university hospitals by a group of experts within the German Medical Informatics Initiative (MII).

Locally adapting the international standard, the Genomics Reporting IG, a FHIR^®^ specification was to be created to provide the dataset definition with a structured exchange format. To show stakeholders how the FHIR profiles could be used to model data contained in German MolGen reports in FHIR^®^, we aimed to create numerous examples as part of the IG. Additional requirements that needed to be integrated to provide greater usability were sought by subjecting the FHIR specification to a public ballot. We also wanted to evaluate the conformance of the dataset with the ISO/TS 20428:2017 standard through a map.

## BACKGROUND AND SIGNIFICANCE

Ever since sequencing technology, particularly next generation sequencing (NGS), has become more accessible and affordable, the integration of genotype, phenotype, and research data has evolved into a primary goal in healthcare.^1^^,^^2^ Assessment of all available patient information, including genomic data and family history of disease, assists in the pursuit of new treatments and a personalized medicine approach.^3^^,^^4^ Oncology and rare diseases are areas where NGS test data is used frequently, primarily to evaluate eligibility for clinical trials and to guide the use of approved therapeutics.^5^ Integration of genetic data with other information in electronic health records (EHR) presents a number of challenges due to their complexity, large volume, and sensitive nature.

A necessary step toward achieving this goal is the compatibility of genomic and phenotype data.^5^^,^^6^ This requires data to be interoperable; compliant with syntactic standards for data structure and a consistent representation of concepts using international classifications and terminologies, such as SNOMED CT^®^, LOINC^®^, and HGVS.^7^ SNOMED CT^®^ is a general purpose classification for coding healthcare data,^8^ whereas the Logical Observation Identifiers Names and Codes (LOINC^®^) terminology is specialized for coding laboratory tests and observations.^9^ The Human Genome Variation Society (HGVS) has developed recommendations for the unambiguous description of sequence variants.^10^^,^^11^

In a medical setting, genomic information is generated through sequencing tests that are performed on a patient’s sample and ordered by the consulting or treating physician. The results of these tests are summarized in a molecular genomics (MolGen) report. Genomic data should be natively stored in a standard-based, interoperable format, otherwise transformation becomes necessary.^12^ In addition to improving clinical decision-making, interoperability enables aggregation of real-world data from various systems across institutional borders which can serve to support research (including big data analytics using artificial intelligence-based methods)^7^ and regulatory decision-making (eg, in the context of drug approvals and or postmarket surveillance of medical devices).^13^

While it is difficult for testing labs and EHRs to implement the ideal state of standardized storage in the short-term, the Health Level 7 (HL7) community has developed the Fast Healthcare Interoperability Resources (FHIR^®^) as a standard layer that helps representing data (including genomic information) in standardized data structures and adopting common terminologies. This approach supports interoperability among various information systems. The building blocks of FHIR are “resources”; data structures that each capture specific data content for a clinical information component (eg, a patient) and define its scope and intended usage. Resources can be adapted to individual use cases through constraints by creating profiles.^14^^,^^15^ HL7’s Clinical Genomics (CG) work group has published the Genomics Reporting IG^16^ as a standard for the structured representation and reporting of variants.

Complementary, the International Organization for Standardization Technical Specification (ISO/TS) 20428 Health informatics standard provides a definition of data fields and metadata necessary to implement a structured clinical genomic sequencing report in EHRs.^17^

In addition, to support the standardized representation and exchange of phenotype information, the Global Alliance for Genomics and Health (GA4GH) has developed a standardized phenopacket data schema.^18^

NGS workflows and approaches to employ data standards, such as FHIR^®^, for the exchange of genomics data have been described in the past.^5^^,^^19^ One of them, Ryu et al,^20^ implemented a structured genomic sequencing report in compliance with ISO/TS 20428 at a tertiary hospital in Korea. However, they had to build numerous resource extensions to cover all required fields. As early adopters of HL7’s Genomics Reporting IG in the United States, Murugan et al, within the Electronic Medical Records and Genomics (eMERGE) network, and Khalifa et al describe similar approaches of identifying key data elements of genetic test reports and mapping them to the IG.^21^ The eMERGE network partners defined 18 core concepts and 100 data elements.^22^

Showing that the international standard is compatible with German genomic reporting requirements, we provide a step-by-step summary of how an exchange format for unstructured genomic report data was created with the aim of achieving integration of genomic and phenotype information in EHRs within the MII^23^ in Germany. Complementing our efforts, partners within the MII have created an openEHR-based genomics model which was also influenced by HL7’s Genomics Reporting IG.^24^ The MII unites all German university hospitals along with other partners in developing tools for the widespread sharing of research results and medical expertise.^8^ One of the MII’s shared goals is the creation of a common HL7 FHIR^®^-based core dataset, structured into informational modules,^25^ the MolGen report being one of them.

Our focus was placed on molecular genomics reporting in the context of oncological and rare disease indications as the majority of sample MolGen reports we reviewed reflected such data. While there are other data models for oncology data in Germany,^26^^,^^27^ ours is the first comprehensive standardized exchange format for genomic data. In fact, four national projects have expressed interest in adopting our FHIR specification. The German Network for Personalized Medicine (DNPM), the genomDE initiative, as well as the HerediVar project of the German Consortium Hereditary Breast and Ovarian Cancer (GC-HBOC) could use our specification in the context of oncology, whereas the Fair4Rare project could adopt it for reporting of variants that contribute to the presence of rare diseases (see Supplementary Materials for project details).

To drive forward the integration of personalized medicine into healthcare in the EU, Germany, along with 21 other EU member countries, have signed on to the “1+ Million Genomes” Initiative, aimed at sequencing a million genomes by 2022.^28^ In this context, reports such as ours could make a significant contribution in increasing the use of healthcare standards for genomic data.

## MATERIALS AND METHODS

### Requirements analysis and dataset definition

The MII set up a working group of around 15 experts from several German university hospitals/research institutions with training in relevant disciplines (detailed for key subject matter experts in Supplementary Table S1). As part of the first step, the dataset definition, the working group reviewed sample MolGen reports anonymized and provided by clinicians at partnering institutions and built on the efforts described by Radke et al.^29^ Of these sample reports, the majority focused on oncological indications, although reports with rare disease indications were considered as well (Supplementary Tables S2 and S3). In an iterative process, the working group identified key concepts (data elements) that altogether comprised a MolGen report and assigned relevance to each data element (labeling it as required, optional or not relevant) and arrived at an agreed-upon dataset definition. In the following step, a logical data model was developed and published on the open-source ART-DECOR^®^ platform^30^ and translated into a UML diagram (Supplementary Figure S1). The output of each step was validated through discussion within the working group, open review by other MII consortia members and approved by the MII’s national steering committee.^31^

### FHIR^®^ specification

In the third step, we used the Genomics Reporting IG, Version 2.0.0,^16^ developed by the HL7 Clinical Genomics work group, as a blueprint for building FHIR^®^ profiles to represent the previously defined MolGen report data elements. All profiles were created using the command-line compiler for FHIR shorthand, SUSHI^32^ and published on Simplifier^®^^33^ and GitHub.^34^

#### Public ballot

The FHIR^®^ specification for the MolGen report was subjected to a public ballot from July 20, 2022 to August 31, 2022. The MII’s project management team sent out an email to all stakeholders asking for comments which could be submitted either by email, or as issues on Simplifier^®^^35^ or GitHub.^36^ For transparency reasons, comments received by email were also posted as issues on GitHub.^36^

### Mapping to ISO/TS 20428:2017

Furthermore, we created a mapping between the MolGen report definition and the required fields defined in the ISO/TS 20428:2017 standard.

## RESULTS

**Figure 1.**
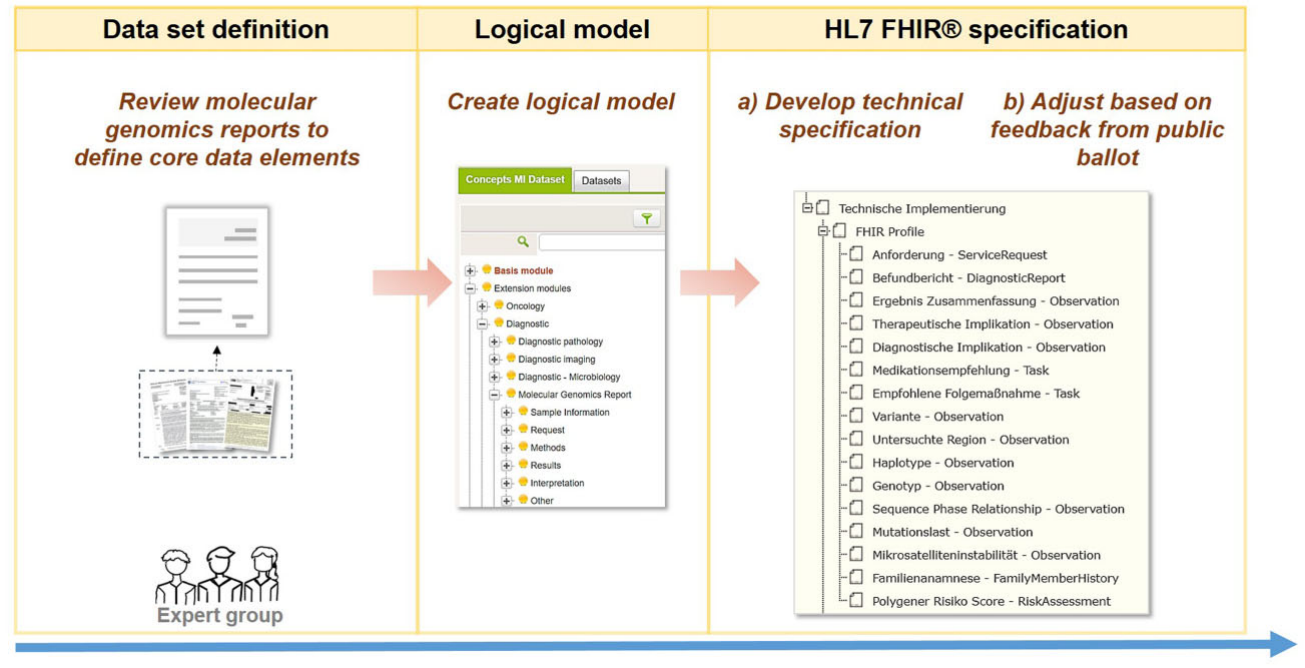
Overview of the steps undertaken in the process of developing a FHIR-based molecular genomics report format.

The 3 steps (requirements analysis and dataset definition, creation of a logical model and FHIR^®^ specification) that we followed to create a FHIR^®^-based MolGen report format within the MII are illustrated in Figure 1.

### Requirements analysis and dataset definition

Over 41 sample MolGen reports from 7 German university hospitals/institutions were analyzed to define the MolGen dataset. The reports covered rare disease (3) and oncological indications (38). Within the oncology category of sample reports reviewed, malignancies that affected 14 different organs were included. Among them, the largest number of reports described malignancies in breast and ovary (12), lung (11), and colorectum (4) (Supplementary Table S3). The reviewed sample reports reflected variants detected in a total of 20 unique genes across all reports (Supplementary Table S4).

**Figure 2.**
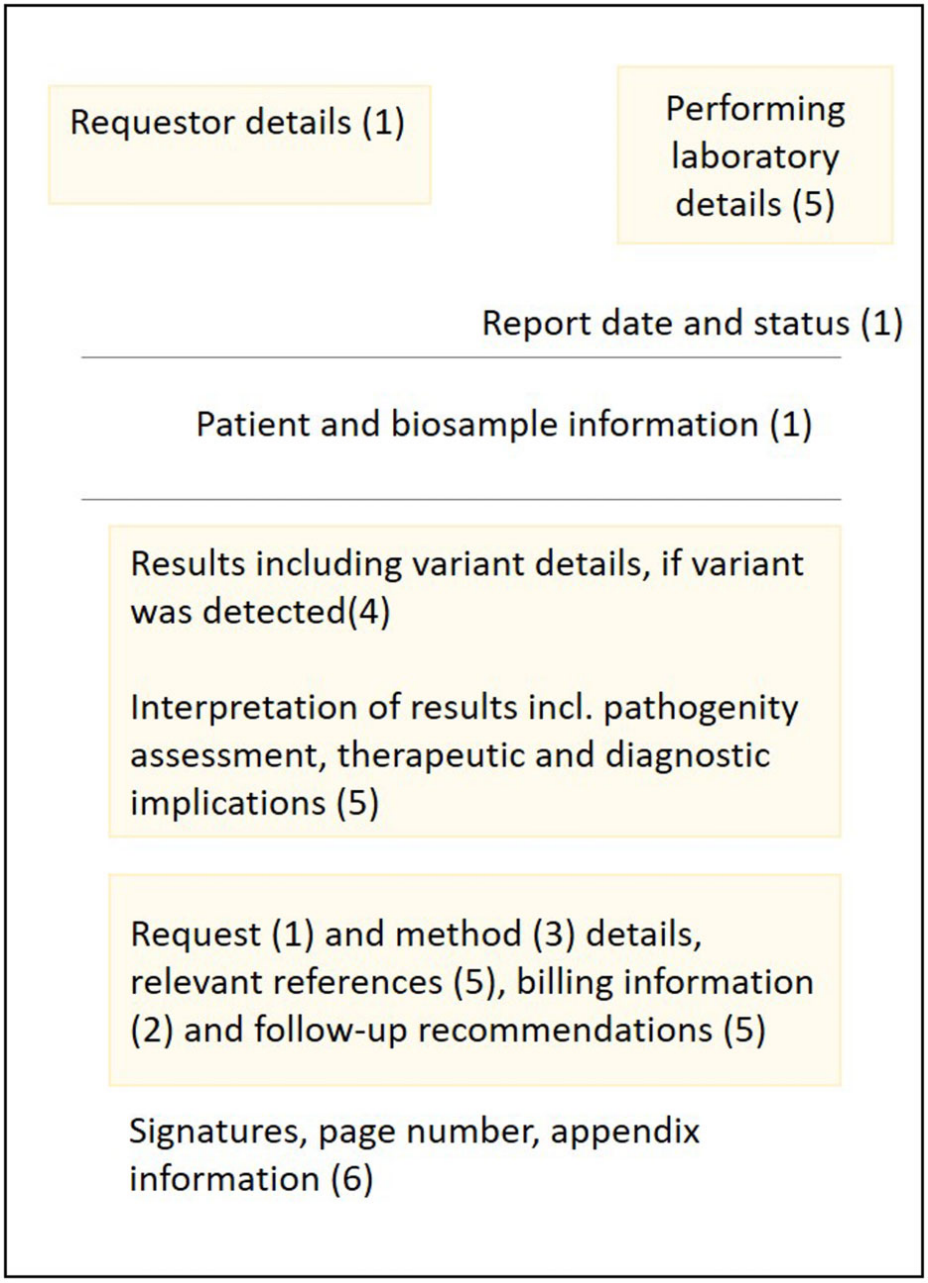
General outline of the main informational elements contained in molecular genomics reports issued by MII project partners. The number in brackets indicates the category of the dataset definition where the respective information was assigned: 1: Specimen information, 2: Request, 3: Methods, 4: Results, 5: Interpretation, 6: Miscellaneous.

The dataset definition, including information about data type, cardinality, and mapping to the corresponding FHIR^®^ element, is shown in Supplementary Table S5. MolGen report information is clustered into 6 categories which are detailed in the following sections and shown in Figure 2.

#### Specimen

The first dataset category includes 11 elements describing specimen characteristics such as specimen type, collection time, and site and additives added during sample processing. A specimen is a human sample provided to the test performing laboratory for analysis. It may be derived from “normal” or from “abnormal” tissue, such as tumor biopsies and also come in form of body fluids. Patient information is also part of the “Specimen” category and covers five data elements that reflect basic personal information (such as name, age, and administrative gender).

#### Request

The request for testing, the requester and supporting information are covered across 14 data elements within the “Request” category. Medical histories of patient and family member(s)’ (if relevant) as well as billing information were incorporated into the “Request” category. Our use case requires incorporation of codes from the “German Uniform Assessment Standard” (German: “Einheitlicher Bewertungsmaßstab,” short EBM). The EBM is the German medical billing fee scale within the national statutory health insurance system.^37^

#### Methods

Ten data elements fall into the third category that focuses on the methods employed. It encompasses the sequencing device, as well as performance characteristics such as read depth, coverage, and detection limit.

#### Results

Results of the performed genomic testing are described across 20 data elements. The majority of them provide information on observed change(s), including DNA and genomic DNA change(s), as well as protein-level change(s). The reviewed sample reports covered somatic and germline variants, hence variant origin was defined as another data element in “Results.” Furthermore, the category also includes the data elements variant ID and cytogenetic location of a variant.

#### Interpretation

The dataset captures the interpretation of the results in 9 data elements. The interpretation includes the clinical significance of the detected variant(s), as well as clinical annotation level of evidence, associated phenotype information, and recommendations. Recommendations are split into three data elements: medication accessed, medication recommendations, and general recommendations can be used to elaborate on the diagnostic and therapeutic implications of the observed genomic characteristics.

#### Miscellaneous

Lastly, 8 data elements fall into the sixth dataset category which focuses on miscellaneous information, such as formal aspects of the report and contact details for the test performing laboratory.

### FHIR^®^ specification

Each element in the MolGen dataset definition was mapped to the appropriate FHIR^®^ element, using the Genomics Reporting IG as a guide.

Table 1 provides an overview of the 14 profiles that were developed for the MII’s FHIR specification for a MolGen report. It also includes a map of short names (aliases) given to the German profile names to simplify references to them in this study.

**Table 1.** Overview of the MII’s MolGen report FHIR^®^ specification profiles and respective short names in English (aliases)

Profile name (German)	Alias (English)	Profile URL
MII PR MolGen Diagnostische Implikation	Diagnostic Implication	https://simplifier.net/packages/de.medizininformatikinitiative.kerndatensatz.molgen/1.0.0/files/803775
MII PR MolGen Familienanamnese	Family Medical History	https://simplifier.net/packages/de.medizininformatikinitiative.kerndatensatz.molgen/1.0.0/files/803772
MII PR MolGen Genotyp	Genotype	https://simplifier.net/packages/de.medizininformatikinitiative.kerndatensatz.molgen/1.0.0/files/803773
MII PR MolGen Medikationsempfehlung	Medication Recommendation	https://simplifier.net/packages/de.medizininformatikinitiative.kerndatensatz.molgen/1.0.0/files/803779
MII PR MolGen Mikrosatelliteninstabilität	Microsatellite Instability	https://simplifier.net/packages/de.medizininformatikinitiative.kerndatensatz.molgen/1.0.0/files/803780
MII PR MolGen Molekulargenetischer Befundbericht	MolGen Finding Report	https://simplifier.net/packages/de.medizininformatikinitiative.kerndatensatz.molgen/1.0.0/files/803771
MII PR MolGen Mutationslast	Mutational Burden	https://simplifier.net/packages/de.medizininformatikinitiative.kerndatensatz.molgen/1.0.0/files/803781
MII PR MolGen Polygener Risiko Score	Polygenic Risk Score	https://simplifier.net/packages/de.medizininformatikinitiative.kerndatensatz.molgen/1.0.0/files/803728
MII PR MolGen Empfohlene Folgemaßnahme	Recommended Follow-Up	https://simplifier.net/packages/de.medizininformatikinitiative.kerndatensatz.molgen/1.0.0/files/803776
MII PR MolGen Untersuchte Region	Region Studied	https://simplifier.net/packages/de.medizininformatikinitiative.kerndatensatz.molgen/1.0.0/files/803783
MII PR MolGen Anforderung genetischer Test	Request	https://simplifier.net/packages/de.medizininformatikinitiative.kerndatensatz.molgen/1.0.0/files/803774
MII PR MolGen Ergebnis Zusammenfassung	Result Summary	https://simplifier.net/packages/de.medizininformatikinitiative.kerndatensatz.molgen/1.0.0/files/803778
MII PR MolGen Therapeutische Implikation	Therapeutic Implication	https://simplifier.net/packages/de.medizininformatikinitiative.kerndatensatz.molgen/1.0.0/files/803782
MII PR MolGen Variante	Variant	https://simplifier.net/packages/de.medizininformatikinitiative.kerndatensatz.molgen/1.0.0/files/803784

Must support flags were set in 14 profiles based on the MII’s analysis requirements. Search parameters were also listed in the implementation guide. We defined 7 additional *SearchParameter* resources to allow search on the:

- “ServiceRequest.reasonCode” and “ServiceRequest.reasonReference” elements in the Request profile, since the reason for molecular testing was critical information to be able to extract from datasets.
- “FamilyMemberHistory.reasonCode” and “FamilyMemberHistory.reasonReference” elements in the Family Medical History profile so that relevant information possibly impacting a patient’s genomic susceptibility to disease could be referenced.
- “Task.for,” “Task.reasonCode,” and “Task.reasonReference” elements in the Recommended Follow-Up and Medication Recommendation profiles so that searches could be performed on the key information in recommendations.

To highlight the intended use of the FHIR specification, we developed resource bundles representing the information contained in 5 sample MolGen reports. These examples reflect anonymized data from actual MolGen reports following cancer-related indications and the suspected presence of rare disease.

#### Sample information

The subsection of the “Specimen” dataset category focusing on the patient’s biological sample is modeled in FHIR using the *Specimen* resource. It is based on the MII’s recently developed “Biobank” module’s FHIR^®^ specification.^38^ In addition, the patient details are modeled after the MII’s “Person” module’s FHIR^®^ specification using the *Patient* resource.^39^ The “Person” module follows the German FHIR^®^ Base specification to represent an individual’s name, address, gender, and relevant personal identifiers.^40^

#### Request

The “Request” dataset category is modeled using a profile of the FHIR Core *ServiceRequest* resource.^41^ The request can be based on (reasonCode) prior testing reports (such as the MII’s “Pathology report” or “Laboratory report” modules^42^^,^^43^) and also reference (reasonReference) observed symptoms. The Phenotypic Feature observation profile defined in GA4GH’s phenopackets IG^44^ can be used to point to symptoms. A practical example of how to do so is included in the MII’s MolGen IG.^45^

**Figure 3.**
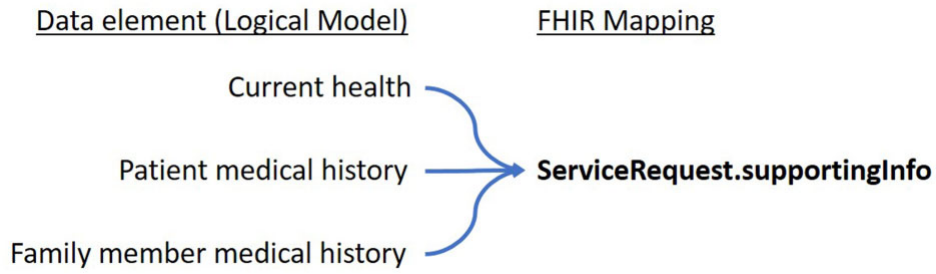
Overview of data elements defined in the logical model of the MII’s MolGen report that can be referenced using the ServiceRequest.supportingInfo extension.

Within the *ServiceRequest,* supporting information (supportingInfo) about the current health status and medical history of the subject/patient can be provided. Our FHIR^®^ specification contains a profile on the *FamilyMemberHistory* resource that can be used to provide familial disease history also via the supportingInfo extension (Figure 3).

To ensure the use of standard terminologies to describe conditions that family members might suffer from, we restricted the condition.code element within the Family Medical History profile to create a slice that allows for the provision of SNOMED CT^®^ codes,^8^ International Classification of Diseases, 10th revision, German modification (ICD-10-GM) codes,^46^ Alpha IDs,^47^ and or Orphanet codes.^48^

The German use case requires the ability to record billing information defined in the EBM using the *ChargeItem* resource, which is referenced using the workflow-supportingInfo extension of the *DiagnosticReport* resource.

#### Methods

The “Methods” section of the dataset can be represented by elements in the Variant, Genotype, Haplotype, Region Studied, and MolGen Finding Report FHIR profiles. The employed method and device can be coded in the Variant profile. An example device has been created and can be reviewed in our IG.^49^ Other characteristics of the method (such as selection of primers, etc.) that are part of the dataset definition were also mapped to elements in the Variant profile.

#### Results

“Results” provide a summary of findings, specifically observed changes (sequence variations), which were mapped primarily to the Region Studied and Variant profiles.

HL7’s Clinical Genomics work group created numerous components within the Variant profile on the *Observation* resource that facilitate the description of observed variations and related information. The Sequence Variant Nomenclature (HGVS)^10^ was defined as the terminology required for coding these changes (on genome-, RNA-, and protein levels).

We extended the Observation.component backbone element in the Variant profile for detailing the detection-limit of the method applied.

### Interpretation

Data elements in the “Interpretation” section of the dataset were mapped to elements specified in the Diagnostic Implication, Therapeutic Implication, Medication Recommendation, Recommended Follow-Up, and Result Summary profiles.

The overall interpretation of detected variant(s) also includes recommendations. Observation.component: medication-assessed and Task.code in the Therapeutic Implication Profile are used to describe medication recommendations while general diagnostic recommendations are mapped to Task.code in the Diagnostic Implication profile.

### Miscellaneous

The “Miscellaneous” section of the dataset covers elements of the MolGen Finding Report profile that provide information about the report ID (identifier), status (status) date (issued), and possible attachments. Depending on the type of attachment provided, the element DiagnosticReport.media or the extension DiagnosticReport.extension: genomics-file^50^ could be selected. The section also includes details on the performer of the requested tests, which can be an organization (referencing the *Organization* resource) or an individual (referencing the *Practitioner* resource).

#### Public ballot

An overview of the comments (issues) that we have received during the ballot phase is shown in Table 2.

**Table 2.** Overview of issues submitted during the balloting phase on the MII MolGen GitHub Repository and on the dedicated IG section in Simplifier^®^

Issue category	GitHub	Simplifier^®^
Dataset/general textual description in IG on Simplifier	1 (closed)	8 (closed)
Understanding of dataset or FHIR specification	5 (closed)	1 (closed)
Specific new requests	2 (open)	1 (closed)
Total issues received	8	10

The majority of the issues (8) opened on the Simplifier^®^ platform highlighted necessary changes to the textual description of the MolGen Report module within the IG. In contrast, most of the issues opened on GitHub included questions regarding the modeling of the metadata in FHIR^®^ to which explanations could be given.

Based on one of the comments, we added a written recommendation in the Variant and Genotype profiles to use the NCBI’s code system (OID urn: oid: 2.16.840.1.113883.6.335) which is based on the International System for Human Cytogenetic Nomenclature (ISCN)^51^ for describing the cytogenetic location of a variant, using the element Observation.component: cytogenetic-location.

**Figure 4.**
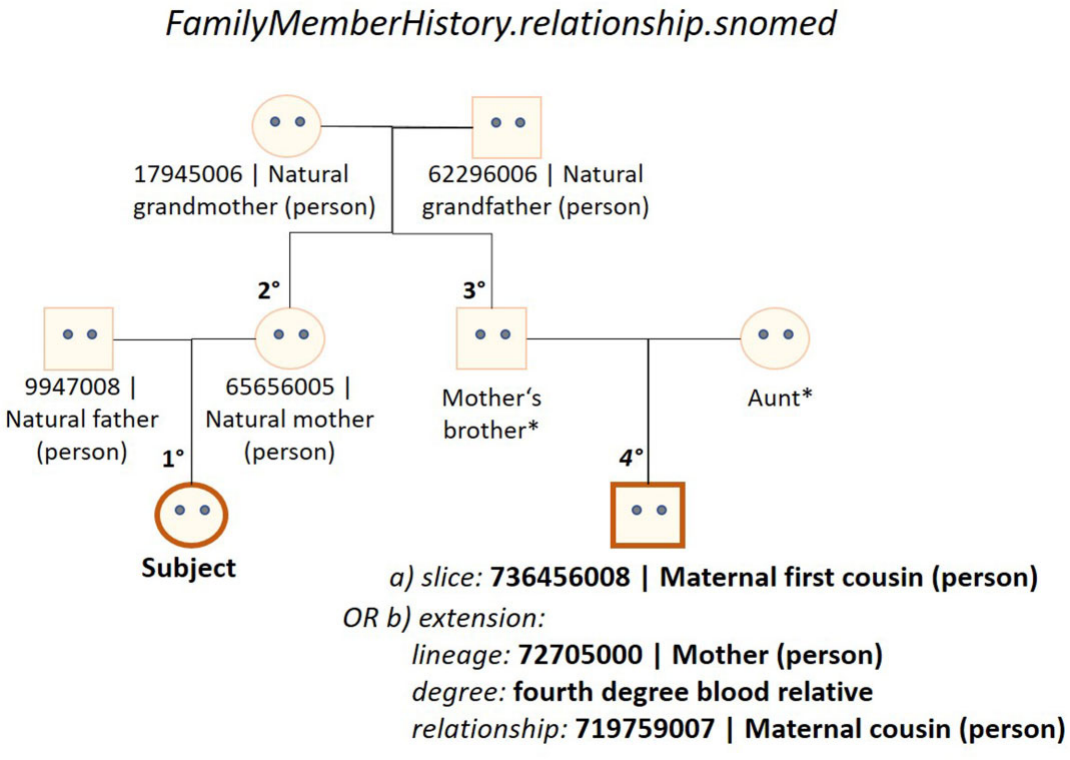
Overview of how the FamilyMemberHistory.relationship.snomed element was extended to enable coding of lineage, degree, and relationship using the SNOMED CT^®^ terminology. *SNOMED CT concepts are currently only available for first and second degree blood relatives but have been requested for third and fourth degree relative concepts.

We received a request to adjust the coding of the relationship element in the Family Medical History profile so that family lineage (detailing whether the relative is a blood relation of the patient’s mother or father), degree of relationship (first degree: 50% shared DNA, second degree: 25% shared DNA, etc.), and type of relationship itself (eg, parent, sibling, etc.) could be coded separately. This is because some MII partners record relationship information in a single concept, others split it into the 3 components, as shown in Figure 4. We created an extension on the relationship data element to enable its specification using either values from the v3.RoleCode^52^ or the SNOMED CT^®^ FamilyMember^53^ value sets.

SNOMED CT^®^ codes describing the relationship and relationship degree were only available for first and second degree. Hence, we opened a request for the creation of third and fourth degree relationship concepts which was submitted to the German release center.^54^

Furthermore, we created a Polygenic Risk Score profile on the *RiskAssessment* resource to facilitate reporting of polygenic risk scores, also requested by MII partners during the ballot phase. A polygenic risk score for an individual represents an estimate of their genetic risk for a trait, typically a disease, based on their genotype. The score takes into consideration effect sizes of many common variants in the genome, often obtained by genome-wide association studies, and aggregates and quantifies them into a score that is reflective of the individual’s genetic risk for a certain disease.^55^ Within the prediction element, the risk score can be entered as a probability score or qualitative risk, whose value can be further restricted by values provided in the when[x] element. Our use case required the ability to add ethnicity of the subject as a relevant influence factor^56^ on the polygenic risk prediction. Hence we created an extension to the prediction.when[x] element so that information that has an impact on the prediction (value of) could be listed. In addition, we have requested the creation of a LOINC^®^ term to represent the polygenic risk score concept.

### Mapping to ISO/TS 20428:2017

We were able to map 30 MolGen report data elements to the required fields listed in the ISO/TC 20428 standard. However, none of these elements were modeled as required in our FHIR^®^ specification. Four required fields noted in the ISO standard are not part of our dataset definition: order received date, addendum creation date, subject of care ethnicity, and medical specialty of ordering physician. An overview of the mapping is provided in Supplementary Table S6.

## DISCUSSION

To achieve interoperability goals, it was required to identify the data elements contained in a MolGen report as a starting point. This approach would eventually lead to avoiding PDF-based information exchange for genomics use cases and facilitate the integration of phenotype and genomic data within EHR systems. In an iterative process, reviewing over 40 sample reports focusing on oncology and rare disease indications, 76 data elements were identified, clustered into 6 categories and put into relation to one another by building an information model. This approach followed the mandated procedural steps of the MII for developing data modules extending or comprising the MII’s core dataset. While 76 elements are significantly more than most other MII modules contain, similar approaches in other countries have identified a comparable number of elements that constitute a MolGen report.^20^ In the next step, we created an exchange standard for the dataset definition that incorporates standard terminologies and classifications such as the use of LOINC^®^ terms to describe laboratory measurements (ie, sequencing) and HGVS codes for variant reporting.

We built on HL7’s Genomics Reporting IG and adapted the profiles for our dataset. Although the international standard covered the majority of data elements defined by our approach, providing supplemental details on billing information, family members’ disease history, and genetic risk scores were also highly relevant for the two use cases that our FHIR specification focuses on; oncological and rare disease indications.^57–59^ Thus, medical history information of family members can be recorded in the Request profile to provide relevant context to the patient’s clinical indication. Condition.code allows for precise coding of diseases through use of ICD-10, ORPHA codes, or Alpha IDs in accordance with recommendations developed in the national project “Rare diseases coding”.^60^

Furthermore, if genomic variations are detected by testing and the evidence suggests the presence of a disease or disorder, the Diagnostic Implication profile allows reference to the applicable polygenic risk score (PRS) profiled in the *RiskAssessment* resource. PRS are thought to partially capture a person’s susceptibility to disease and are reported as auxiliary information in some of the sample MolGen reports we reviewed. The clinical utility of the score still needs to be fully ascertained and will likely require more data.^61^

The mapping against the required and optional fields defined within the ISO/TS 20428 standard was performed to evaluate compliance of our dataset with the standard. The results highlight the fact that there is still significant heterogeneity in how genomic sequencing findings are reported within MolGen reports in Germany which is the reason why we could not make all defined data elements mandatory to use. Ryu et al^20^ show they were able to implement the required elements within one hospital.

### Limitations

FHIR^®^ resources have been designed to meet the 80:20 rule,^62^^,^^63^ addressing 20% of necessary specifications that meet 80% of interoperability needs. The current version of our FHIR^®^ specification for a MolGen report supports the structured representation of single nucleotide variants (SNVs), copy number variants (CNVs), and DNA fusions. It does not facilitate the structured representation of gene expression levels (ie, detected by fluorescence in situ hybridization, FISH^64^), although this was requested by stakeholders. Likewise, the specifications still has to be expanded to enable structured reporting of:

- complex variants, such as gene fusions (RNA) with the necessary level of detail, and
- processing steps (bioinformatics pipeline).

We are in active discussion with members of HL7’s CG work group to develop consensus models for these data elements. Moreover, the current data model was developed taking into review only 3 sample MolGen reports with rare disease indications. Further adaptations might become necessary in order to accommodate the rare disease use-case more precisely.

### Outlook

Genomic testing (data) needs to be integrated into IT systems to enable automatic ordering of tests and use of the generated data for clinical decision support and research. Developing SMART on FHIR-based applications for genomic test data is one possible solution.^65^ Hence, the next crucial step to advance the broad integration of standardized electronic molecular genomics reporting into clinical practice in Germany would be to increase the availability and use of FHIR-based laboratory applications. Williams et al have reported on applying a “Software as a Service” (SaaS) approach to build a platform for the clinical display and exchange of genomic test reports, however without employing common exchange formats. Several other studies also detail approaches of using computerized information retrieval tools, referred to as “infobuttons,” to develop genetic reporting applications that can be integrated into EHR systems to give healthcare providers access to genetic test results at the point of care.^66–68^ Dolin et al^69^ recently reported on the use of so called Genomics Operations that extend FHIR query capabilities to simplify access to genomics data to facilitate clinical decision support.

## CONCLUSION

We have created a dataset definition and an information model comprising of 76-information items that captures all genomic and supplemental information contained in a MolGen report generated by university hospitals within the MII in Germany. In addition, to achieve compatibility with phenotype data and integration into EHR systems, a locally customized adoption of HL7’s Genomics Reporting IG as a standard format for exchanging MolGen reports was developed. The FHIR^®^ specification includes profiles on the *FamilyMemberHistory* and *RiskAssessment* resources and also contains 5 resource bundles as examples of how MolGen report data can be modeled in FHIR. This effort reflects the requirements of and will support various German initiatives (DNPM, HerediVar, genomDE) for interoperable health data.

## Supplementary Material

ocad061_Supplementary_Data

## Data Availability

The logical model is publicly available for viewing on the ART-DECOR^®^ platform (https://art-decor.org/ad/#/mide-/datasets/dataset/). The current built (version: 1.0.0) of the information model together with the corresponding implementation guide is hosted on the Simplifier^®^ platform, and also accessible to the public. A dedicated GitHub Repository contains all IG files (https://github.com/medizininformatik-initiative/kerndatensatzmodul-GenetischeTests).
