# Supplementary material for: Creation of a structured molecular genomics report for Germany as a local adaption of HL7’s Genomic Reporting Implementation Guide
Source: J Am Med Inform Assoc. 2023 Apr 20;30(6):1179–89. doi: 10.1093/jamia/ocad061 (PMC10198526; doi:10.1093/jamia/ocad061)
Supplement: ocad061_Supplementary_Data [file ocad061_supplementary_data.docx]

# SUPPLEMENTARY MATERIAL

### German Medical Informatics Initiative (MII)

The MII has been developing a core data set using international IT (HL7 FHIR) and terminology standards, as a prerequisite for data sharing. The information covered by the data set must be recorded at minimum by all partners for consented in-patients and are stored at each data institution’s data integration center. The data set comprises of base modules that are not specific to any particular medical discipline and generic in nature, and extension modules that are specific to certain disciplines, such as microbiology, pathology or molecular genetics[1].

### Four possible future adopters (use-cases) of the MolGen FHIR specification are:

#### DNPM

The Federal Joint Committee (G-BA) is funding the German Network for Personalized Medicine (short DNPM) project from 2021-2025. The project aims at bundling the competencies of 21 university hospitals with a comprehensive cancer center (CCC) in personalized medicine to develop common standards and institute molecular tumor boards in a harmonized center structure, the so called centers for personalized medicine (ZPM), to provide patients with personalized treatment solutions, focusing on cancer in a first step[2].

#### GenomDE

In October 2021, the German Federal Ministry of Health started funding the ‘Initiative for the establishment of a nation-wide platform for medical genome sequencing’ (short genomDE). The 14-member consortium is tasked with developing a nation-wide platform to facilitate greater clinical use of genome sequencing in the context of personalized medicine with the ultimate goal of better diagnosis and treatment identification[3].

#### GC-HBOC

One of the partners in the genomeDE initiative is the German Consortium Hereditary Breast and Ovarian Cancer (GC-HBOC) which connects 23 university hospitals and 244 cooperation partners in their goal to create a knowledge-generating care concept for risk-adapted breast and ovarian cancer prevention[4]. The GC-HBOC is leading the HerediVar project, which is funded by the German Cancer Aid to develop bioinformatics tools for the classification of genetic variants in ovarian and breast cancer[5].

#### FAIR4Rare

The FAIR4Rare project will receive funding from the German Federal Joint Committee to support its intent of developing a National Registry for Rare Diseases (NARSE)[6].

| Supplementary Table 1: Overview of the qualifications of key subject matter experts involved in the creation of the MolGen report data model. |
| --- |
| \| **Expert name** \| **Current title  (Affiliation)** \| **Years of relevant experience** \| **Field of expertise** \| \| --- \| --- \| --- \| --- \| \| Dr. B. Auber \| Team Lead, High Throughput Sequencing Unit, Department of Human Genetics  (Medizinische Hochschule Hannover) \| 20 \| Human genetics \| \| Prof. Dr. M. Boeker \| Professor of Medical Informatics, Institute of Artificial Intelligence and Informatics in Medicine (Technical University of Munich) \| 20 \| Medical informatics, Artificial intelligence \| \| Prof. Dr. Dipl.-Phys. F. Klauschen \| Associate Director, Institute of Pathology, (Charité - Universitätsmedizin Berlin) Director, Institute of Pathology, (Ludwig-Maximilians-Universität München) Fellow (The Berlin Institute for the Foundations of Learning and Data (BIFOLD)) \| 15 \| Human genetics, Pathology \| \| Prof. Dr. R. Schmutzler \| Director, Center for Familial Breast and Ovarian Cancer (Universitätsklinikum Köln) \| 30 \| Oncology, Gynaecology \| \| Prof. Dr. S. Thun \| Director, Core-Unit eHealth and Interoperability  (Berlin Institute of Health at Charité - Universitätsmedizin Berlin) \| 20 \| Digital medicine, Interoperability \| \| Prof. Dr. T. Wienker \| Senior Research Fellow  (Max Planck Institute for Molecular Genetics) \| 30 \| Molecular human genetics \| |

| Supplementary Table 2: Number of sample molecular genomics reports received by the MII working group to aid the data set definition, listed by the institution that provided the report(s). |
| --- |
| \| **Institution** \| **Number of reports** \| \| --- \| --- \| \| Charité - Universitätsmedizin Berlin \| 2 \| \| Ludwig-Maximilians-Universität München (LMU) \| 2 \| \| Medizinische Hochschule Hannover \| 3 \| \| Medizinisches Versorgungszentrum am Städtischen Klinikum Karlsruhe GmbH \| 1 \| \| NCT Heidelberg \| 6 \| \| Universitätsklinikum Köln \| 24 \| \| Universitätsklinikum Tübingen \| 3 \| \| **Total** \| **41** \| |

| Supplementary Table 3: Overview of the affected organs mentioned in the reviewed sample molecular genomics reports that were provided to the MII working group by partnering institutions. |
| --- |
| \| **Affected organ mentioned in report** \| **Number of reports** \| \| --- \| --- \| \| Abdomen and stomach \| 2 \| \| Adrenal gland \| 1 \| \| Bile duct \| 1 \| \| Bladder \| 1 \| \| Breast and Ovary \| 12 \| \| Colorectal \| 4 \| \| Endometrium \| 1 \| \| Eye \| 1 \| \| Lung \| 11 \| \| Skin \| 1 \| \| Soft tissue \| 1 \| \| Thyroid \| 1 \| \| Multiple organs (Rare disease) \| 3 \| \| Systemic \| 1 \| |

| Supplementary Table 4: Overview of genes in which variants were detected as listed in the reviewed sample molecular genomics reports. |
| --- |
| \| **Variant Gene(s)** \| **Oncology** \| **Rare disease** \| \| --- \| --- \| --- \| \| *No variant detected* \| 5 \| 1 \| \| ATM \| 1 \|  \| \| BRAF \| 3 \|  \| \| BRCA1 \| 6 \|  \| \| BRCA1, BRAF, PTEN, SMO, TERT, CDKN2A \| 1 \|  \| \| BRCA2 \| 2 \|  \| \| CTNNA1 \| 1 \|  \| \| CTNNB1, GREB1 \| 1 \|  \| \| EGFR \| 4 \|  \| \| EGFR, TP53 \| 1 \|  \| \| ERBB2, KRAS \| 1 \|  \| \| FGFR1 \| 1 \|  \| \| FGFR2, TP53 \| 1 \|  \| \| KRAS \| 2 \|  \| \| KRAS, TP53 \| 2 \|  \| \| MET \| 1 \|  \| \| NIPBL \|  \| 1 \| \| NR4A2 \| 1 \|  \| \| NRAS \| 1 \|  \| \| PRPF31 \|  \| 1 \| \| RET \| 1 \|  \| \| SF3B1 \| 1 \|  \| \| TP53 \| 1 \|  \| \| **Total** \| **38** \| **3** \| |

| Supplementary Table 5: Overview of the data set definition for the German MolGen report developed within the MII and mapping of each data element to FHIR elements, as reflected in the MolGen report implementation guide.   \| **Category** \| **Data set element** \| **Data type** \| **Cardinality** \| **FHIR mapping** \| **Profile name (alias)** \| \| --- \| --- \| --- \| --- \| --- \| --- \| \| **(1) Specimen information** \| \|  \|  \|  \|  \| \| ***Patient*** \| Patient-ID \| Identifier \| 0..* \| ServiceRequest.subject(Patient.identifier) \| Request \| \|  \| Name \| HumanName \| 1..* \| ServiceRequest.subject(Patient.name) \| \|  \| Administrative gender \| Extension.valueCoding \| 1..1 \| ServiceRequest.subject(Patient.gender) \| \|  \| Date of birth \| date OR DateTime \| 1..1 \| ServiceRequest.subject(Patient.birthDate) \| \|  \| Address \| Address \| 1..* \| ServiceRequest.subject(Patient.address) \| \| ***Specimen*** \| Specimen-ID \| Identifier \| 0..* \| ServiceRequest.specimen(Specimen.identifier) \| \|  \| Specimen type \| CodeableConcept \| 1..1 \| ServiceRequest.specimen(Specimen.type) \| \|  \| Collection site \| CodeableConcept \| 0..1 \| ServiceRequest.specimen(Specimen.collection.bodySite) \| \|  \| Shape \| Annotation \| 0..* \| ServiceRequest.specimen(Specimen.note) \| \|  \| Specimen amount \| SimpleQuantity \| 0..1 \| ServiceRequest.specimen(Specimen.collection.quantity) \| \|  \| Additive \| Reference \| 0..* \| ServiceRequest.specimen(Specimen.processing.additive) \| \|  \| Quantitiy of additive/solvent \| Reference \| 0..1 \| ServiceRequest.specimen(Specimen.processing.additive(Substance.ingredient.quantity)) \| \|  \| Tumor/neoplastic cell percentage \|  \|  \|  \| \|  \| Time of collection \| Period OR dateTime \| 1..1 \| ServiceRequest.specimen(Specimen.collection.collected[x]) \| \|  \| Specimen receipt date \| dateTime \| 0..1 \| ServiceRequest.specimen(Specimen.receivedTime) \| \|  \| Other characteristics \| Annotation \| 0..* \| ServiceRequest.specimen(Specimen.note) \| \| **(2) Request** \|  \|  \|  \|  \|  \| \| ***Indication*** \| Indication \| CodeableConcept \| 0..* \| ServiceRequest.reasonReference OR ServiceRequest.reasonCode \| Request \| \|  \| Current health \| Reference \| 0..* \| ServiceRequest.supportingInfo(Condition) \| \|  \| Family member medical history \| Reference \| 0..* \| ServiceRequest.supportingInfo(FamilyMemberHistory) \| \|  \| Carrier of genetic disposition \| Reference \| 0..* \| ServiceRequest.supportingInfo(FamilyMemberHistory), ServiceRequest.supportingInfo(Condition), ServiceRequest.supportingInfo(Observation) \| \|  \| Relevant prior results \| Reference \| 0..* \| ServiceRequest.reasonReference(Condition), ServiceRequest.reasonReference(Observation), ServiceRequest.reasonReference(DiagnosticReport), ServiceRequest.reasonReference(DocumentReference) \| \| ***Requester*** \| Name \| HumanName CodeableConcept string \| 0..* \| ServiceRequest.requester(Practioner.name), ServiceRequest.requester(PractionerRole.code) ServiceRequest.requester(Organization.name) \| \|  \| Address \| Address \| 0..* \| ServiceRequest.requester(Practioner.address), ServiceRequest.requester(Organization.address) \| \|  \| Contact details \| ContactPoint \| 0..* \| ServiceRequest.requester(Practioner.telecom), ServiceRequest.requester(PractionerRole.telecom), ServiceRequest.requester(Organization.telecom) \| \| ***Request*** \| Genes to be tested \| CodeableConcept \| 0..* \| ServiceRequest.code \| \|  \| German Uniform Assessment Standard \| Reference \| 0..* \| DiagnosticReport.extension:supportingInfo(ChargeItem) \| MolGen Finding Report \| \|  \| Request text \| string \| 0..1 \| ServiceRequest.code.text \| Request \| \|  \| Request date \| dateTime \| 0..1 \| ServiceRequest.authoredOn \| \|  \| Note \| Annotation \| 0..1 \| ServiceRequest.note \| \| **(3) Methods** \|  \|  \|  \|  \|  \| \|  \| Method \| CodeableConcept \| 0..1 \| Observation.method \| Variant, Genotype \| \|  \| Relevant parameters \| Reference \| 0..* \| DiagnosticReport.extension:supporting-info(Observation), DiagnosticReport.extension:supporting-info(DocumentReference) \| MolGen Finding Report \| \|  \| Devices / Software / Kits \| Reference \| 0..1 \| Observation.device \| Variant, Genotype \| \|  \| Tested genes \| CodeableConcept \| 0..* \| Observation.component:gene-studied \| Variant, Haplotype, Genotype \| \|  \| Reference sequence \| CodeableConcept \| 0..1 \| Observation.component:transcript-ref-seq \| Variant, Region Studied \| \|  \| Read depth / Coverage \| CodeableConcept \| 0..1 \| Observation.component:allelic-read-depth \| \|  \| Intron spanning / IVS \| string \| 0..1 \| Observation.component:dna-region \| Variant \| \|  \| Start and end nucleotide \| Range \| 0..1 \| Observation.component:exact-start-end \| \|  \| Sensitivity /detection limit \| Quantity \| 0..1 \| Observation.component:detection-limit \| \|  \| Limitations / Note \| Annotation \| 0..* \| Observation.note \| \| **(4) Results** \|  \|  \|  \|  \|  \| \|  \| Summary \| CodeableConcept \| 0..1 \| Observation.value \| Result Summary \| \| ***Changes*** \| Change on protein level \| CodeableConcept \| 0..1 \| Observation.component:protein-hgvs \| Variant \| \|  \| DNA changes \| CodeableConcept \| 0..1 \| Observation.component:coding-hgvs \| \|  \| Genomic DNA change \| CodeableConcept \| 0..1 \| Observation.component:genomic-hgvs \| \|  \| Transcript ID \| CodeableConcept \| 0..1 \| Observation.component:transcript-ref-seq \| \|  \| Reference genome \| CodeableConcept \| 0..* \| Observation.component:reference-sequence-assembly \| Variant, Genotype \| \|  \| Alt allele \| CodeableConcept \| 0..1 \| Observation.component:alt-allele \| Variant \| \|  \| Ref allele \| CodeableConcept \| 0..1 \| Observation.component:ref-allele \| \|  \| DNA mutation type \| CodeableConcept \| 0..1 \| Observation.component:coding-change-type \| \|  \| Mutation consequence (functional) \| CodeableConcept \| 0..1 \| Observation.component:amino-acid-change-type \| \|  \| Sample allelic frequency \| CodeableConcept \| 0..1 \| Observation.component:sample-allelic-frequency \| \|  \| Variant origin \| CodeableConcept \| 0..1 \| Observation.component:genomic-source-class \| \|  \| Variant ID \| CodeableConcept \| 0..* \| Observation.component:variation-code \| \|  \| Chromosome \| CodeableConcept \| 0..* \| Observation.component:chromosome-identifier \| \|  \| Exon \| CodeableConcept \| 0..* \| Observation.component:dna-region \| \|  \| Cytogenetic location \| CodeableConcept \| 0..* \| Observation.component:cytogenetic-location \| Variant, Haplotype, Genotype \| \|  \| Copy number variation \| CodeableConcept \| 0..1 \| Observation.component:copy-number \| Variant \| \|  \| Mutational burden \| Quantity \| 0..1 \| Observation.valueQuantity \| Mutational Burden \| \|  \| Microsatellite instability \| CodeableConcept \| 0..1 \| Observation.valueCodeableConcept \| Microsatellite Instability \| \|  \| Data \| Reference \| 0..* \| DiagnosticReport.extension:genomics-file(DocumentReference) \| MolGen Finding Report \| \| **(5) Interpretation** \|  \|  \|  \|  \|  \| \|  \| Clinical significance \| CodeableConcept \| 0..1 \| Observation.component:clinical-significance \| Diagnostic Implication \| \|  \| References \| RelatedArtifact \| 0..1 \| Observation.extension:relatedArtifact \| \|  \| Clinical annotation levels of evidence \| CodeableConcept \| 0..* \| Observation.component:evidence-level \| Diagnostic Implication, Therapeutic Implication \| \|  \| Associated phenotype \| CodeableConcept \| 0..* \| Observation.component:predicted-phenotype, Observation.component:phenotypic-treatment-context \| \|  \| Mode of inheritance \| CodeableConcept \| 0..1 \| Observation.component:mode-of-inheritance \| Diagnostic Implication \| \|  \| Summary \| CodeableConcept \| 0..1 \| Observation.component:conclusion-string \| Diagnostic Implication, Therapeutic Implication, Result Summary \| \|  \| Medication assessment \| CodeableConcept \| 0..* \| Observation.component:medication-assessed \| Therapeutic Implication \| \|  \| Recommendations \| CodeableConcept \| 1..1 \| Task.code \| Recommended Follow-up \| \|  \| Medication recommendation \| CodeableConcept \| 1..1 \| Task.code \| Medication Recommendation \| \| **(6) Miscellaneous** \|  \|  \|  \|  \|  \| \|  \| Report ID \| Identifier \| 0..* \| DiagnosticReport.identifier \| MolGen Finding Report \| \|  \| Attachment \| Reference \| 0..* \| DiagnosticReport.extension:genomics-file DiagnosticReport.media \| \|  \| Report status \| code \| 1..1 \| DiagnosticReport.status \| \|  \| Report date \| instant \| 0..1 \| DiagnosticReport.issued \| \| ***Laboratory / Institution / Contact person*** \| Performer accreditations \| BackboneElement \| 0..* \| DiagnosticReport.performer(Practioner.qualification) DiagnosticReport.performer(PractionerRole.qualification) DiagnosticReport.performer(Organization.qualification) \| \|  \| Performer name \| HumanName string \| 0..* 0..1 \| DiagnosticReport.performer(Practioner.name) DiagnosticReport.performer(PractionerRole.name) DiagnosticReport.performer(Organization.name) \| \|  \| Performer address \| Address \| 0..* \| DiagnosticReport.performer(Practioner.address) DiagnosticReport.performer(PractionerRole.address) DiagnosticReport.performer(Organization.address) \| \|  \| Performer contact \| ContactPoint \| 0..* \| DiagnosticReport.performer(Practioner.telecom) DiagnosticReport.performer(PractionerRole.address) DiagnosticReport.performer(Organization.telecom) \| |
| --- | --- | --- | --- | --- | --- | --- | --- | --- | --- | --- | --- | --- | --- | --- | --- | --- | --- | --- | --- | --- | --- | --- | --- | --- | --- | --- | --- | --- | --- | --- | --- | --- | --- | --- | --- | --- | --- | --- | --- | --- | --- | --- | --- | --- | --- | --- | --- | --- | --- | --- | --- | --- | --- | --- | --- | --- | --- | --- | --- | --- | --- | --- | --- | --- | --- | --- | --- | --- | --- | --- | --- | --- | --- | --- | --- | --- | --- | --- | --- | --- | --- | --- | --- | --- | --- | --- | --- | --- | --- | --- | --- | --- | --- | --- | --- | --- | --- | --- | --- | --- | --- | --- | --- | --- | --- | --- | --- | --- | --- | --- | --- | --- | --- | --- | --- | --- | --- | --- | --- | --- | --- | --- | --- | --- | --- | --- | --- | --- | --- | --- | --- | --- | --- | --- | --- | --- | --- | --- | --- | --- | --- | --- | --- | --- | --- | --- | --- | --- | --- | --- | --- | --- | --- | --- | --- | --- | --- | --- | --- | --- | --- | --- | --- | --- | --- | --- | --- | --- | --- | --- | --- | --- | --- | --- | --- | --- | --- | --- | --- | --- | --- | --- | --- | --- | --- | --- | --- | --- | --- | --- | --- | --- | --- | --- | --- | --- | --- | --- | --- | --- | --- | --- | --- | --- | --- | --- | --- | --- | --- | --- | --- | --- | --- | --- | --- | --- | --- | --- | --- | --- | --- | --- | --- | --- | --- | --- | --- | --- | --- | --- | --- | --- | --- | --- | --- | --- | --- | --- | --- | --- | --- | --- | --- | --- | --- | --- | --- | --- | --- | --- | --- | --- | --- | --- | --- | --- | --- | --- | --- | --- | --- | --- | --- | --- | --- | --- | --- | --- | --- | --- | --- | --- | --- | --- | --- | --- | --- | --- | --- | --- | --- | --- | --- | --- | --- | --- | --- | --- | --- | --- | --- | --- | --- | --- | --- | --- | --- | --- | --- | --- | --- | --- | --- | --- | --- | --- | --- | --- | --- | --- | --- | --- | --- | --- | --- | --- | --- | --- | --- | --- | --- | --- | --- | --- | --- | --- | --- | --- | --- | --- | --- | --- | --- | --- | --- | --- | --- | --- | --- | --- | --- | --- | --- | --- | --- | --- | --- | --- | --- | --- | --- | --- | --- | --- | --- | --- | --- | --- | --- | --- | --- | --- | --- | --- | --- | --- | --- | --- | --- | --- | --- | --- | --- | --- | --- | --- | --- | --- | --- | --- | --- | --- | --- | --- | --- | --- | --- | --- | --- | --- | --- | --- | --- | --- | --- | --- | --- | --- | --- | --- | --- | --- | --- | --- | --- | --- | --- | --- | --- | --- | --- | --- | --- | --- | --- | --- | --- | --- | --- | --- | --- | --- | --- | --- | --- | --- | --- | --- | --- | --- | --- | --- | --- | --- | --- | --- | --- | --- | --- | --- | --- | --- | --- | --- | --- | --- | --- | --- | --- |

| Supplementary Table 6: Mapping performed between the MolGen report data set definition and the required elements described in ISO/TC 20428. |
| --- |
| \| **ISO/TC 20248 field category** \| **ISO/TC 20248 field** \| **Included in MII MolGen report?** \| **MII MolGen report data element** \| **Cardinality in MII MolGen report** \| \| --- \| --- \| --- \| --- \| --- \| \| **Clinical sequencing orders** \| \|  \|  \|  \| \|  \| Order code \| yes \| Request text \| 0..1 \| \|  \| Information on sequencing order \| yes \| Request text \| 0..*;  0..1 \| \|  \| Order date \| yes \| Request date \| 0..1 \| \|  \| Order received date \| no \|  \|  \| \|  \| Report date \| yes \| Report date \| 0..1 \| \|  \| Addendum creation date \| no \|  \|  \| \|  \| Specimen information \| yes \| Specimen-ID \| 0..* \| \| **Information on subject of care** \| \|  \|  \|  \| \|  \| Subject of care identifiers \| yes \| Patient: Patient-ID \| 0..* \| \|  \| Subject of care name \| yes \| Patient: Name \| 0..* \| \|  \| Subject of care birth date \| yes \| Patient: Date of birth \| 0..1 \| \|  \| Subject of care sex \| yes \| Patient: Administrative gender \| 0..1 \| \|  \| Subject of care ethnicity \| no \|  \|  \| \| **Information on legally authorized person ordering clinical sequencing** \| \| \|  \|  \| \|  \| Name of ordering physician \| yes \| Requester: Name \| 0..1 \| \|  \| Medical specialty of ordering physician \| no \|  \| 0..* \| \|  \| Contact numbers \| yes \| Requester: Contact \| 0..* \| \|  \| Address \| yes \| Requester: Address \| 0..* \| \| **Performing laboratory** \| \|  \|  \|  \| \|  \| Basic information on performing laboratory \| yes \| Laboratory / Institution / Contact person \| 0..* \| \|  \| Information on report generator \| yes \| Laboratory / Institution / Contact person \|  \| \|  \| Information of legally confirmed person on sequencing report \| yes \| Laboratory / Institution / Contact person \|  \| \| **Associated diseases and phenotypes** \| \|  \|  \|  \| \|  \| *Diseases or phenotypes associated with found variants* \| yes \| Associated phenotype \| 0..* \| \| **Biomaterial information** \| \|  \|  \|  \| \|  \| Type of sample \| yes \| Specimen type \| 1..1 \| \|  \| Genomic source class of sample \| yes \| Variant origin \|  \| \|  \| Conditions of specimen that may limit adequacy of testing \| yes \| Other characteristics \| 0..* \| \| **Genetic variations** \| \|  \|  \|  \| \|  \| Gene symbols and names \| yes \| Methoden.Getestete Gene ID \| 0..* \| \|  \| ***Sequence variation information*** \|  \|  \|  \| \|  \| HGVS nomenclature \| yes \| Change on protein level \| 0..1 \| \|  \| DNA changes \| \|  \| Genomic DNA change \| \|  \| Effects of variants \| yes \| Mutation consequence (functional) \| 0..1 \| \|  \| Sequence variant ID \| yes \| Variant ID \| 0..* \| \| **Classification of variants** \| \|  \|  \|  \| \|  \| Classification of variants based on the pathogeny \| yes \| Clinical Annotation Level of Evidence \| 0..* \| \|  \| Classification of variants based on clinical relevance \| yes \| Clinical significance \| 0..1 \| \| **Recommended Treatment** \| \|  \|  \|  \| \|  \| Medication \| yes \| Medication recommendation \| **1..1** \| \|  \| Clinical Trial Information \| yes \| Recommendations \| 0..1 \| \|  \| Known protocols related to a variant \| yes \| References \| 0..1 \| \|  \| Other recommendation \| yes \| Recommendations \| 0..1 \| \| **Addendum** \| \| yes \| Attachment \| 0..* \| |

| 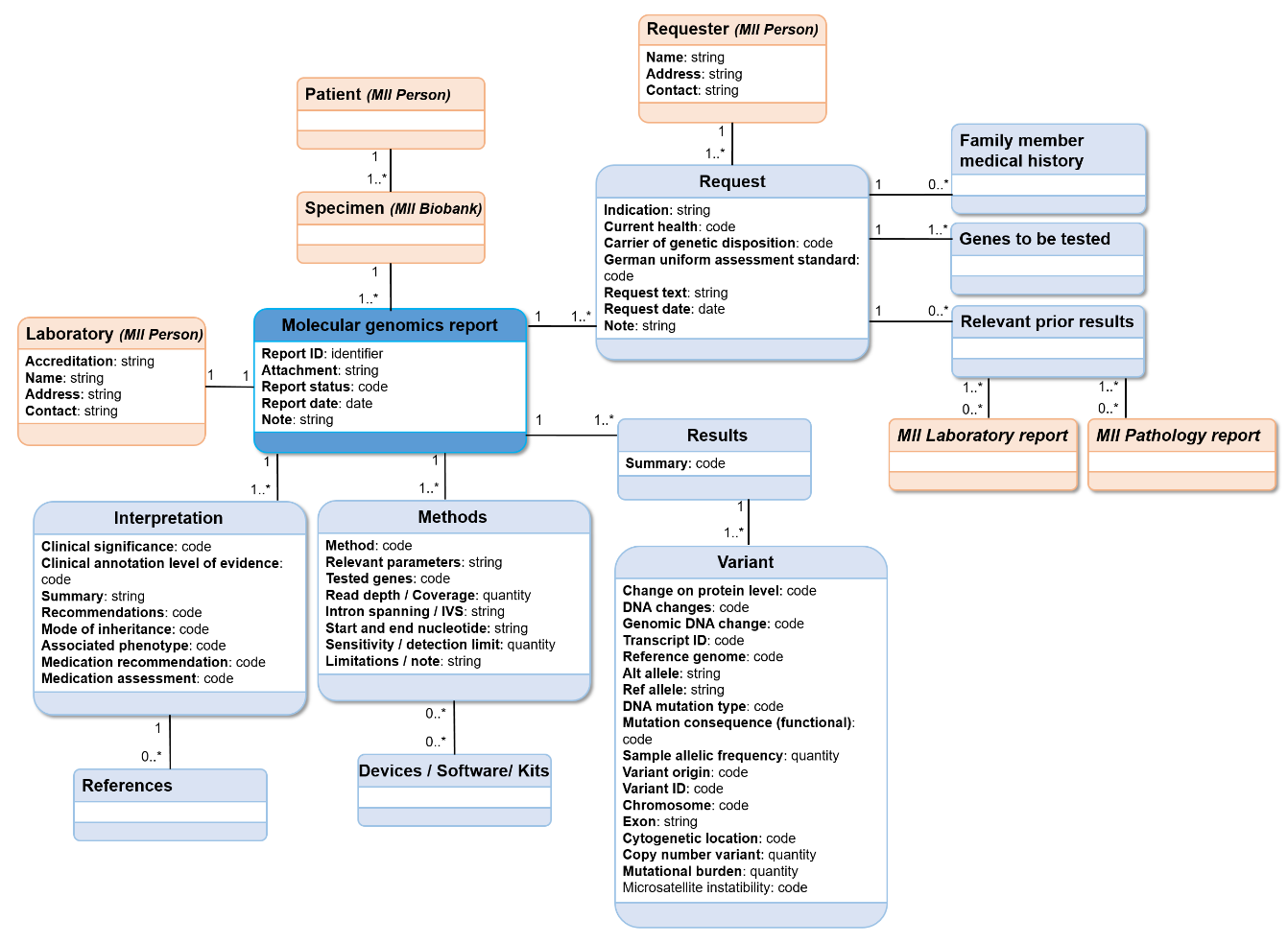 |
| --- |
| Supplementary Figure 1: Unified Modeling Language diagram created for the MII’s MolGen report data set definition. |

# References

[1] S. C. Semler, F. Wissing, and R. Heyder, “German Medical Informatics Initiative: A National Approach to Integrating Health Data from Patient Care and Medical Research,” *Methods Inf. Med.*, vol. 57, no. Suppl 1, p. e50, Jul. 2018, doi: 10.3414/ME18-03-0003.

[2] German Cancer Society, “Health Services Research.” https://www.krebsgesellschaft.de/gcs/german-cancer-society/certification/health-services-research.html (accessed Nov. 29, 2022).

[3] Bundesministerium für Gesundheit, “Die deutsche Genom-Initiative - genomDE,” Jul. 14, 2022. https://www.bundesgesundheitsministerium.de/themen/gesundheitswesen/personalisierte-medizin/genomde-de.html (accessed Nov. 03, 2022).

[4] Rita Schmutzler, “Das Deutsche Konsortium Familiärer Brust- und Eierstockkrebs DK-FBREK.” Nov. 30, 2020. Accessed: Nov. 13, 2022. [Online]. Available: https://www.bundesgesundheitsministerium.de/fileadmin/Dateien/3_Downloads/G/genomDE/Dokumente/7-genomDE_Conference_Prof.Dr.Schmutzler_FBREK.pdf

[5] K. Rhiem *et al.*, “Consensus Recommendations of the German Consortium for Hereditary Breast and Ovarian Cancer,” *Breast Care*, vol. 17, no. 2, pp. 199–207, Apr. 2022, doi: 10.1159/000516376.

[6] E. Thull, “‘Entscheidung mit Weitsicht’ - Eva Luise und Horst Köhler Stiftung,” Jun. 27, 2022. https://www.elhks.de/en/innovationsfonds-foerdert-fair4rare/ (accessed Nov. 29, 2022).
